# Advancements and expanding applications of CAR-T cell therapy

**DOI:** 10.3389/fimmu.2026.1802718

**Published:** 2026-03-24

**Authors:** Xiang Zhang, Zi-Chang Liu, Ling-jie Luo, Yan-wei Wu, Xin-Gang Cui, Liang Chen

**Affiliations:** 1 School of Medicine, Shanghai University, Shanghai, China; 2 Institute of Artificial Intelligence and Biomanufacturing, School of Medicine, Shanghai University, Shanghai, China; 3 Shanghai Tenth People’s Hospital, Shanghai, China; 4 Department of Urology, Xinhua Hospital, Shanghai Jiao Tong University School of Medicine, Shanghai, China

**Keywords:** autoimmune diseases, CAR-T cell therapy, gene editing, infectious diseases, universal CAR-T cells

## Abstract

CAR T-cell therapy has become a transformative modality in oncology, demonstrating sustained clinical efficacy in hematologic malignancies. Recent investigations have expanded its potential beyond cancer to immune-mediated disorders, including autoimmune diseases such as multiple sclerosis and systemic lupus erythematosus, as well as chronic viral infections including HIV and hepatitis B. This review examines the mechanistic foundations of CAR-T cells, advances in universal and allogeneic engineering strategies designed to mitigate graft-versus-host disease and host rejection, and emerging *in vivo* gene-delivery platforms that aim to bypass conventional ex vivo manufacturing. We further evaluate safety-control architectures, including logic-gated and inducible systems, and discuss translational barriers related to scalability, manufacturing standardization, and long-term immune durability. While technological innovations in genome editing, synthetic biology, and computational design continue to refine CAR-T platforms, substantial biological and logistical challenges remain. A critical synthesis of these evolving strategies is necessary to distinguish incremental optimization from paradigm-shifting advances and to define the future trajectory of CAR-based immunotherapy across oncology and immune-mediated diseases.

## 1 Introduction

Chimeric Antigen Receptor (CAR) T-cell therapy has ushered in a revolutionary era in cancer treatment, particularly for hematological malignancies where conventional therapies have often fallen short. The FDA-approved therapies, such as tisagenlecleucel for acute lymphoblastic leukemia, have demonstrated remarkable success, leading to long-lasting remissions and offering new hope for patients with treatment-resistant cancers (1–3).

However, the application of CAR-T therapy in solid tumors remains challenging. Despite substantial advances, CAR-T efficacy in solid tumors remains constrained by immunosuppressive microenvironments, intratumoral antigen heterogeneity, and the scarcity of tumor-restricted targets (4, 5).

The limitations encountered in solid tumors have stimulated growing interest in extending CAR-T therapy beyond oncology, particularly to diseases where conventional treatments remain suboptimal, including autoimmune disorders and chronic viral infections. However, expanding CAR-T applications into these areas introduces additional biological complexity. In hematologic malignancies, therapeutic success is partly facilitated by the accessibility of circulating tumor cells and the relative uniformity of lineage-restricted antigens such as CD19 (6). In contrast, autoimmune diseases and chronic infections are characterized by intricate immune regulatory networks, tissue-specific immune compartmentalization, antigen variability, and dynamic immune adaptation (7, 8). In these contexts, effective therapy requires not only potent cytotoxic activity but also precise control of persistence, immune homeostasis, and long-term immune reconstitution.

Recent research has highlighted the adaptability of CAR-T technology, offering promise for treating immunological disorders like multiple sclerosis and systemic lupus erythematosus (9). Unlike traditional therapies that use broad immunosuppressants, CAR-T cells can be engineered to specifically target aberrant immune cells or inflammatory mediators, offering a more precise therapeutic approach (10). Furthermore, innovations in genetic engineering, including the development of universal CAR-T cells and advanced gene delivery systems, are enhancing the feasibility and safety of CAR-T therapies, thereby broadening their potential applications (11).

Although CAR-T therapy has achieved remarkable breakthroughs, the field has rapidly diversified into multiple engineering paradigms, delivery strategies, and disease applications, resulting in conceptual and translational fragmentation. Critical questions remain unresolved: which universal CAR-T approaches are truly scalable? Can *in vivo* engineering realistically replace ex vivo manufacturing, or will it remain complementary? How can safety-control mechanisms be incorporated without diminishing therapeutic efficacy? A rigorous synthesis of these approaches is necessary to clarify the field’s trajectory and to differentiate incremental refinements from transformative innovations. This review provides a comparative and critical evaluation of CAR-T strategies across oncology, autoimmune diseases, and chronic infections, highlighting both technological advances and persistent challenges.

## 2 CAR-T core mechanisms

**Figure 1:**
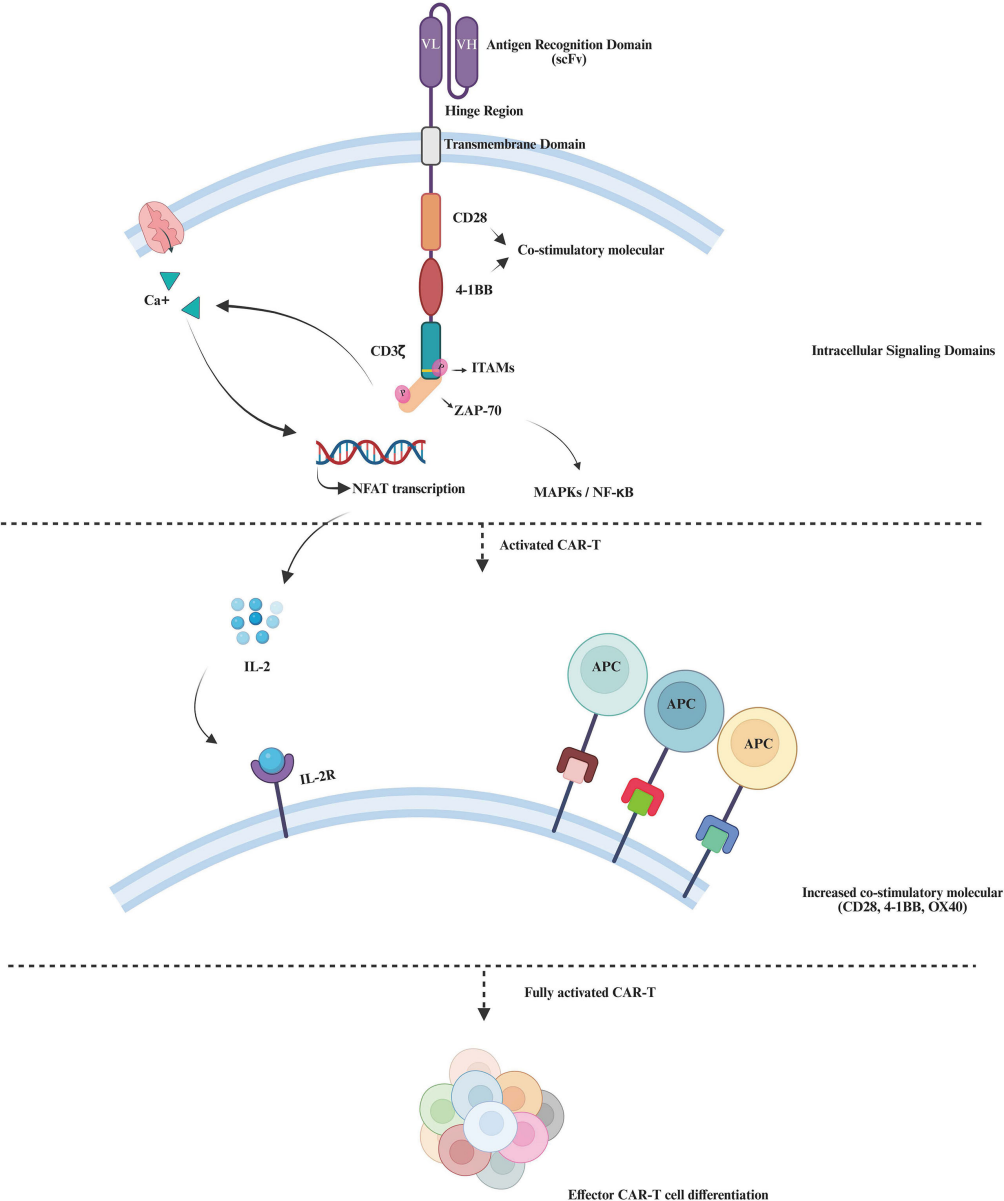
Activation and function of third-generation CAR-T. This figure illustrates the activation and differentiation of third-generation CAR-T cells, from antigen recognition to full activation and effector T-cell differentiation. The CAR structure on the T-cell surface comprises scFv, a hinge region, a transmembrane domain, and intracellular signaling domains including CD3ζ and co-stimulatory molecules (CD28, 4-1BB). Antigen binding initiates a signaling cascade starting with the phosphorylation of ITAMs in the CD3ζ chain, activating ZAP-70, which then increases intracellular Ca^2+^ to support NFAT transcription, essential for T-cell activation. Signal amplification continues through the MAPK and NF-κB pathways, enhancing T-cell gene expression and survival. Subsequent IL-2 production interacts with IL-2R on CAR-T cells, promoting their proliferation and sustained activation. The increased expression of additional co-stimulatory molecules (CD28, 4-1BB, OX40) enhances interactions with APCs, leading to full activation and differentiation of CAR-T cells into various effector types like cytotoxic, memory, and helper T cells, crucial for targeted therapeutic actions. CD3ζ, CD3 Zeta Chain; ITAMs, Immunoreceptor Tyrosine-based Activation Motifs; ZAP-70, Zeta-chain-associated Protein Kinase 70; NFAT, Nuclear Factor of Activated T-cells; MAPK, Mitogen-Activated Protein Kinase; NF-κB, Nuclear Factor Kappa-light-chain-enhancer of activated B cells; IL-2, Interleukin-2; IL-2R, Interleukin-2 Receptor; APCs, Antigen-Presenting Cells.

The core of CAR-T cell therapy centers on its transformative ability to repurpose the body’s immune cells toward targeting and eliminating specific antigens. This therapeutic capacity initiates with a crucial genetic modification step where T cells are engineered to express a chimeric antigen receptor (CAR) (12). The construction of the CAR involves an extracellular antigen recognition domain, typically derived from an antibody’s single-chain variable fragment (scFv), which is essential for recognizing and binding to antigens on the surfaces of target cells (13). This scFv is linked to one or more intracellular signaling domains that are vital for the downstream T-cell activation, proliferation, and survival. These signaling domains include CD3ζ and co-stimulatory molecules like CD28 and 4-1BB, which collectively orchestrate a complex activation cascade (14, 15) detailed in **Figure 1**. This cascade enhances the CAR-T cells’ therapeutic functions, enabling them to effectively identify and destroy malignantly transformed or pathogen-infected cells (16).

Beyond oncological targets, these mechanisms are being explored for their potential to combat immunological disorders through specific antigen targeting. For instance, in autoimmune diseases such as systemic lupus erythematosus and myasthenia gravis, researchers are investigating CAR-T cells designed to target antigens expressed on aberrant immune cells or inflammatory mediators (17, 18). This approach aims to selectively deplete harmful cell populations without broadly suppressing the immune system, potentially offering a more targeted and less toxic alternative to conventional therapies that use broad-acting immunosuppressants (19–21).

## 3 Allogeneic and universal CAR-T strategies

CAR-T therapies can be categorized into autologous and allogeneic platforms. Allogeneic CAR-T cells are derived from healthy donors and developed as “off-the-shelf” products, offering advantages such as rapid availability, standardized manufacturing, and potential cost reduction. However, unmodified allogeneic CAR-T cells carry risks of graft-versus-host disease (GVHD) due to endogenous T-cell receptor (TCR) recognition and host immune rejection caused by HLA mismatch (22).

**Figure 2:**
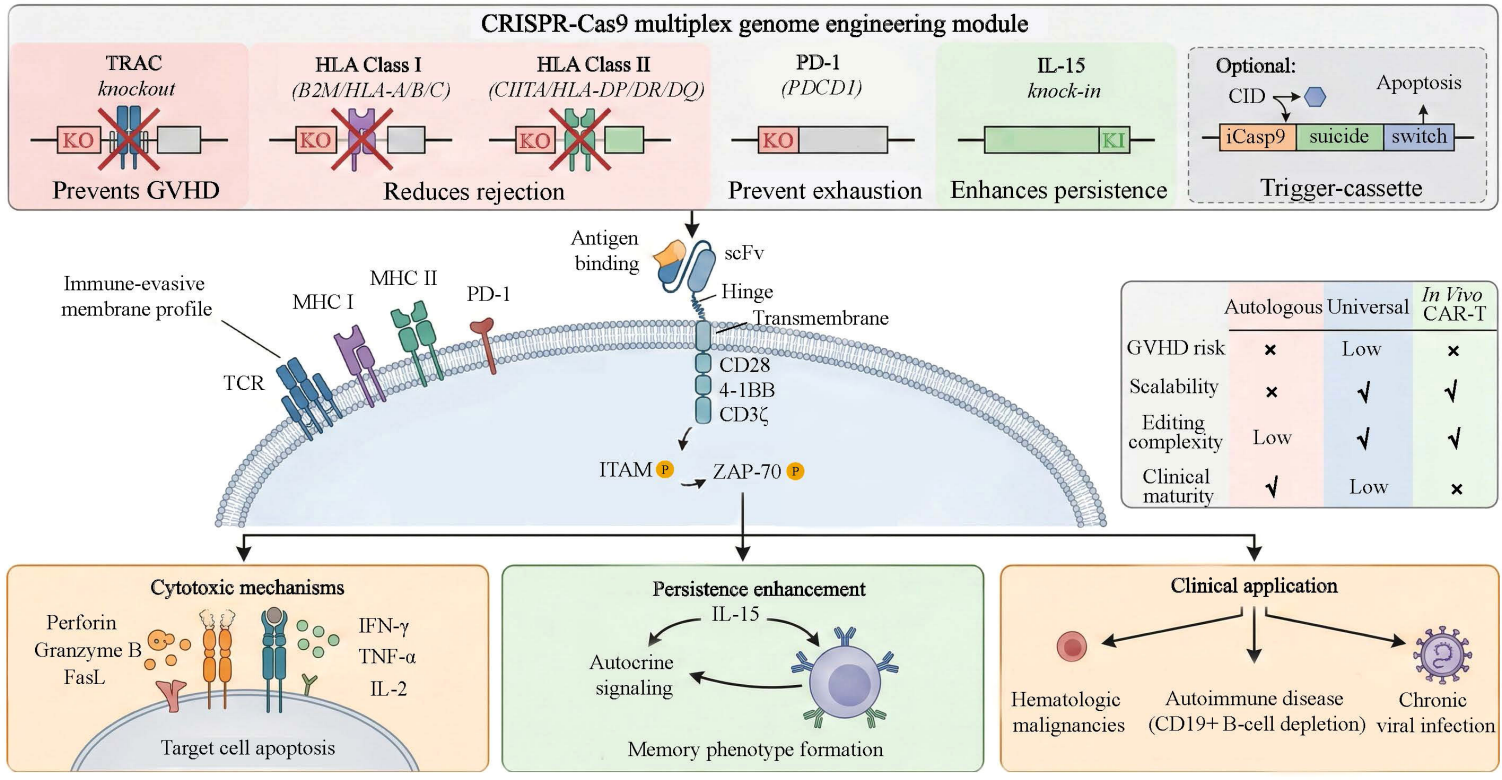
CRISPR–engineered universal CAR-T cell design and functional overview.

Universal CAR-T cells are a genetically engineered subset of allogeneic CAR-T therapies designed to minimize alloreactivity and enable broader applicability. The term “universal” encompasses two distinct strategies (1): gene-edited allogeneic CAR-T cells with disruption of the endogenous T-cell receptor (e.g., TRAC knockout) and HLA modification to reduce GVHD and immune rejection; and (2) modular or switchable CAR-T platforms (e.g., UNICAR) that allow antigen redirection and external control of activity (**Figure 2**). Additional genetic modifications, such as PD-1 disruption or cytokine armoring, may further enhance persistence and efficacy. Importantly, although all universal CAR-T cells are allogeneic, not all allogeneic CAR-T products fulfill the strict definition of universal CAR-T therapy.

Several strategies have been developed to generate universal CAR-T cells. Gene-editing technologies such as TALENs, CRISPR/Cas9, and base editing enable precise disruption of the TCR a chain (TRAC locus) and HLA molecules, thereby reducing the risk of GVHD and rejection. In parallel, multiple clinical trials have evaluated both donor-derived allogeneic CAR-T platforms and gene-edited universal CAR-T products across various malignancies Notably, recent clinical investigations have expanded these strategies beyond oncology to autoimmune and infection diseases.

(**Table 1**).

**Table 1:** Selected clinical trials and reports of allogeneic/universal or broad CAR-T cell therapies in autoimmune and infection diseases.

Clinical trial identifier	Target disease	CAR target	Platform type	Key findings
ChiCTR2100047968	Recurrent High-Grade Gliomas	B7H3	Allogeneic (reported as universal; gene-edited platform)	Evaluated safety, efficacy, and pharmacokinetics; no severe adverse effects; 42.9% response rate (24)
ISRCTN15323014	Relapsed/Refractory AML	CD123	Universal (switchable UNICAR platform)	Phase I dose escalation; mild CRS; encouraging CRi responses (complete remission with incomplete recovery) (25)
ISRCTN15323014	Pediatric Relapsed/Refractory T-ALL	CD7	Gene-edited Universal (base-edited)	Demonstrated feasibility and early anti-leukemic efficacy; manageable safety; 56% response rate (26)
NCT04557436	Pediatric Relapsed/Refractory B-ALL	CD19	CRISPR-engineered Universal	Two of six patients achieved CR with MRD negativity; acceptable safety profile (27)
NCT04093596 (UNIVERSAL trial)	Relapsed/Refractory Multiple Myeloma	BCMA	Allogeneic	55.8% response rate; manageable CRS and neurotoxicity (28)
NCT05716113	Relapsed/Refractory T-ALL/LBL	CD7	Gene-edited Universal	94% CR/CRi rate; 100% MRD negativity in CR/CRi patients; no DLT or neurotoxicity; manageable CRS (29)
NCT05859997	Myositis/Systemic sclerosis	CD19	CRISPR-engineered Universal	Induced clinical remission; manageable toxicity (30)
Clinical report	Refractory autoimmune pediatric conditions	CD19	Autologous	Symptom improvement; organ recovery (31)
Case report	HIV-1 infection	gp120	Autologous	First-in-human feasibility demonstrated (32)

In addition to gene-edited universal approaches, several clinical trials have evaluated donor-derived allogeneic CAR-T products that do not fully disrupt TCR or HLA expression but employ alternative safety strategies. For instance, CYAD-101 utilizes a non-gene-editing platform incorporating a TCR inhibitory strategy to reduce alloreactivity without direct genomic disruption (23). These products represent important advances in allogeneic CAR-T therapy but may not meet strict definitions of “universal” CAR-T cells.

Schematic of a multiplex CRISPR–Cas9 strategy for generating universal CAR-T cells. Knockout (KO) of TRAC prevents GVHD, while disruption of HLA class I (β2M/HLA-A/B/C) and class II (CIITA/HLA-DP/DR/DQ) reduces host rejection. PD-1 (PDCD1) KO limits exhaustion, and IL-15 knock-in enhances persistence and memory formation through autocrine signaling. An optional inducible caspase-9 (iCasp9) suicide switch provides safety control.

The CAR construct includes an scFv antigen-binding domain, hinge, transmembrane region, and intracellular signaling modules that activate ZAP-70–mediated pathways, leading to cytotoxicity (perforin, granzyme B, FasL) and cytokine secretion (IFN-γ, TNF-α, IL-2). A comparative framework outlines differences among autologous, universal, and in vivo CAR-T platforms in terms of GVHD risk, scalability, editing complexity, and clinical maturity.

### 3.1 Comparative evaluation of universal CAR-T engineering strategies

Although multiple engineering strategies have been developed to generate universal CAR-T cells, their relative advantages and translational feasibility differ substantially. Gene-editing approaches such as TRAC disruption, HLA deletion, and base editing aim to reduce alloreactivity with varying degrees of genomic manipulation. In contrast, non-gene-editing platforms such as CYAD-101 attempt to mitigate GVHD without permanent genome alteration. A comparative synthesis of these strategies highlights important trade-offs between safety, scalability, and clinical maturity (**Table 2**).

**Table 2:** Comparative analysis of universal CAR-T platforms.

Strategy	Strength	Major risk	Scalability
TRAC knockout	Reduces GVHD	Rejection via HLA mismatch	Moderate
HLA deletion	Reduces rejection	NK-mediated clearance	Moderate
Base editing	Reduces chromosomal translocations	Limited long-term data	Emerging
Non-editing	Avoids genomic risk	Possibly less durable	Low–moderate

## 4 CAR-T versus CAR-NK cell therapies

CAR-engineered natural killer (CAR-NK) cells have emerged as an important platform in cellular immunotherapy, particularly for allogeneic “off-the-shelf” applications.

CAR-T cells exhibit potent antigen-specific cytotoxicity, robust *in vivo* expansion, and long-term persistence, which underpin their clinical success in hematologic malignancies. However, they are associated with notable toxicities, including CRS, immune effector cell–associated neurotoxicity syndrome (ICANS), and the risk of GVHD in allogeneic settings. In addition, autologous manufacturing remains complex and resource-intensive (33).

CAR-NK cells offer several potential advantages. NK cells possess intrinsic cytotoxicity mediated by germline-encoded activating receptors, enabling tumor killing even in the setting of antigen downregulation (34). They generally produce lower levels of pro-inflammatory cytokines such as IL-6, contributing to a reduced incidence and severity of CRS and neurotoxicity in early clinical studies (35). Importantly, NK cells carry minimal risk of GVHD, supporting their development as universal products derived from peripheral blood, cord blood, NK cell lines, or induced pluripotent stem cells (36).

However, CAR-NK cells typically demonstrate shorter *in vivo* persistence and limited expansion compared with CAR-T cells, which may affect durability of response. Strategies including IL-15 armoring and optimized gene-editing approaches are being investigated to enhance their persistence and anti-tumor efficacy (37).

## 5 *In vivo* CAR-T

*In vivo* CAR-T strategies have attracted growing attention as a potential alternative to conventional ex vivo manufacturing. Unlike traditional ex vivo approaches, *in vivo* methods aim to directly modify T-cells within the patient’s body, offering advantages in scalability and reduced manufacturing complexities. Recent advancements in gene delivery technologies have paved the way for more efficient and targeted *in vivo* CAR-T cell engineering.

### 5.1 LNPs for mRNA or DNA delivery and CRISPR/Cas9 gene editing

Lipid nanoparticles (LNPs) and polymer-based nanoparticles are emerging as promising tools in *in vivo* CAR-T therapy, enabling the direct delivery of CAR-encoding mRNA or DNA to T cells. LNPs can be engineered for targeted delivery, efficiently transfecting T cells to generate CAR-T cells capable of targeting specific antigens, such as CD19 or CD20 (38, 39). In the case of CRISPR/Cas9, LNPs are utilized to deliver the CRISPR components (Cas9 protein and guide RNA) to the T cells, facilitating precise genetic modifications such as the introduction of CAR constructs or enhancements to improve T cell persistence and efficacy (40).

LNPs offer advantages in *in vivo* CAR-T therapy, including simplified production, scalability, and reduced immune response risks. However, LNP technology is largely monopolized by a few companies, which may limit accessibility and increase costs. Additionally, challenges remain in optimizing T cell targeting, ensuring efficient CAR gene expression, and minimizing off-target effects, which could impact the success of CAR-T cell generation.

### 5.2 Viral vectors

In contrast, viral vectors, such as lentiviral vectors or adeno-associated virus (AAV), have gained attention for their ability to efficiently transduce T cells and ensure the stable integration of CAR constructs. For example, systems like TetraVecta have been developed to improve the specificity and efficiency of CAR-T cell generation *in vivo*. These vectors can be targeted to T cells using bispecific binders, ensuring precise delivery of the CAR construct (41).

The primary advantage of viral vectors lies in their high transfection efficiency, which allows for stable and long-term integration of the CAR constructs in T cells, resulting in sustained activity (42). However, viral vectors carry immunogenic risks, as immune responses against viral components could lead to adverse effects. Additionally, the risk of insertional mutagenesis, where the viral DNA integrates into the host genome and potentially causes oncogenic transformation, is a major concern (43). Furthermore, the manufacturing process for viral vectors remains complex and costly, limiting the scalability of this approach (44).

### 5.3 Exosome-based delivery

Exosome-based delivery represents a more novel strategy for *in vivo* CAR-T cell engineering. Exosomes are naturally occurring vesicles secreted by cells, which can be engineered to carry CAR mRNA or proteins. These exosomes are administered to the patient and deliver the CAR components to T cells, facilitating CAR-T cell generation *in vivo*. Research has explored the use of engineered exosomes to carry CAR mRNA and anti-CD3/CD28 single-chain variable fragments (scFvs), enabling the direct conversion of primary T cells to CAR T cells *in vivo* (45). The key advantage of exosome-based delivery lies in its biocompatibility and the ability to avoid the complexities of viral or non-viral gene delivery systems. However, challenges remain in the efficient production of exosomes and ensuring that they deliver the CAR components effectively to T cells.

### 5.4 Bispecific antibody-mediated delivery

Bispecific antibodies are another method used for the *in vivo* generation of CAR-T cells. These antibodies are designed to bind both T cells and the CAR construct, allowing for targeted delivery of the CAR gene to T cells without the need for viral vectors or nanoparticles. Bispecific antibody-redirected lentiviral vectors have shown effectiveness in generating CAR T cells and inducing anti-tumor activity (46). The advantage of bispecific antibodies is their ability to specifically target T cells, reducing the risk of off-target effects. However, the challenge lies in ensuring that these bispecific antibodies are both highly specific and efficient in facilitating the CAR-T cell generation.

### 5.5 Biomimetic scaffolds delivery

Biomimetic scaffolds can be used to directly engineer T cells *in vivo* into CAR-T cells. These scaffolds, made from biodegradable materials, mimic the natural extracellular matrix and provide a supportive environment for T cells to expand, differentiate, and be engineered into CAR-T cells. By incorporating CAR-specific ligands or cytokines, these scaffolds facilitate the direct conversion of native T cells into functional CAR-T cells within the patient’s body, eliminating the need for ex vivo manipulation (47, 48). This approach enhances scalability and reduces the complexities of traditional CAR-T cell therapies, enabling efficient *in vivo* CAR-T cell generation and potentially improving the persistence and efficacy of the therapy. However, challenges remain, including the efficiency of *in vivo* CAR-T engineering, potential immune responses to scaffold materials, and regulatory concerns regarding safety and long-term effects.

Taken together, these emerging *in vivo* engineering strategies represent diverse technological solutions to a shared translational challenge: achieving efficient, safe, and scalable CAR-T generation within the patient. Although viral vectors offer superior transduction efficiency and durable expression, they carry insertional mutagenesis risks and complex manufacturing burdens. In contrast, LNP-based delivery enables scalable production and transient expression but faces challenges in T-cell targeting efficiency. Exosome-based systems may provide improved biocompatibility; however, their production standardization and cargo-loading consistency remain unresolved. At present, no single platform achieves the optimal balance of specificity, durability, safety, and scalability, underscoring the need for hybrid or next-generation delivery strategies.

### 5.6 Challenges and future directions

*In vivo* CAR-T cell generation offers advantages over traditional ex vivo methods, including improved scalability, cost-effectiveness, and accessibility. However, challenges remain in optimizing efficiency, safety, and gene delivery precision. Future research should focus on refining delivery systems (e.g., viral vectors, LNPs, exosomes), regulating immune responses, and improving manufacturing scalability. Combining *in vivo* CAR-T therapies with other treatments, like checkpoint inhibitors, could further enhance their efficacy.

## 6 Comparative framework of CAR-T platforms

CAR-T therapy currently comprises three main paradigms: autologous ex vivo products, allogeneic/universal off-the-shelf platforms, and emerging *in vivo* gene-delivery approaches. While all rely on antigen-specific immune redirection, they differ in scalability, immunological risk, and clinical maturity (**Table 3**).

**Table 3:** Comparative overview of CAR-T engineering paradigms.

Feature	Autologous CAR-T	Allogeneic/universal CAR-T	*In vivo* CAR-T
Cell Source	Patient-derived	Healthy donor–derived	Endogenous T cells
Manufacturing	Individualized ex vivo	Batch-produced ex vivo	No ex vivo expansion
Time to Treatment	Weeks	Days to weeks	Potentially rapid
GVHD Risk	None	Requires TCR disruption	None
Rejection Risk	Minimal	Possible host rejection	Not applicable
Gene Editing Complexity	Low–moderate	High (multiplex editing)	Delivery-dependent
Persistence	High	Variable	Currently limited data
Scalability	Limited	Moderate–high	Potentially high
Clinical Maturity	FDA-approved products	Early-phase trials	Preclinical/early clinical
Cost Structure	Very high	Potentially reduced	Theoretical reduction

Autologous CAR-T is the most validated approach, offering durable persistence without GVHD risk, but it requires individualized manufacturing, resulting in high cost and treatment delay.

Allogeneic platforms improve availability and standardization through donor-derived batch production. However, preventing GVHD and rejection requires complex gene editing, and long-term persistence remains under evaluation.

*In vivo* CAR-T seeks to eliminate ex vivo manufacturing entirely by directly programming endogenous T cells. Although potentially scalable and cost-efficient, this strategy is still early in development and faces delivery and safety challenges.

## 7 Expanding CAR-T cell application beyond blood cancers

CAR-T cell therapy is not only a groundbreaking treatment for blood cancers but also holds promise for addressing a wide range of other medical conditions. Beyond hematologic cancers, its potential is being explored in autoimmune diseases, infectious diseases, and solid tumors, offering targeted treatment options where traditional therapies have often been limited or ineffective.

### 7.1 Autoimmune diseases

**Figure 3:**
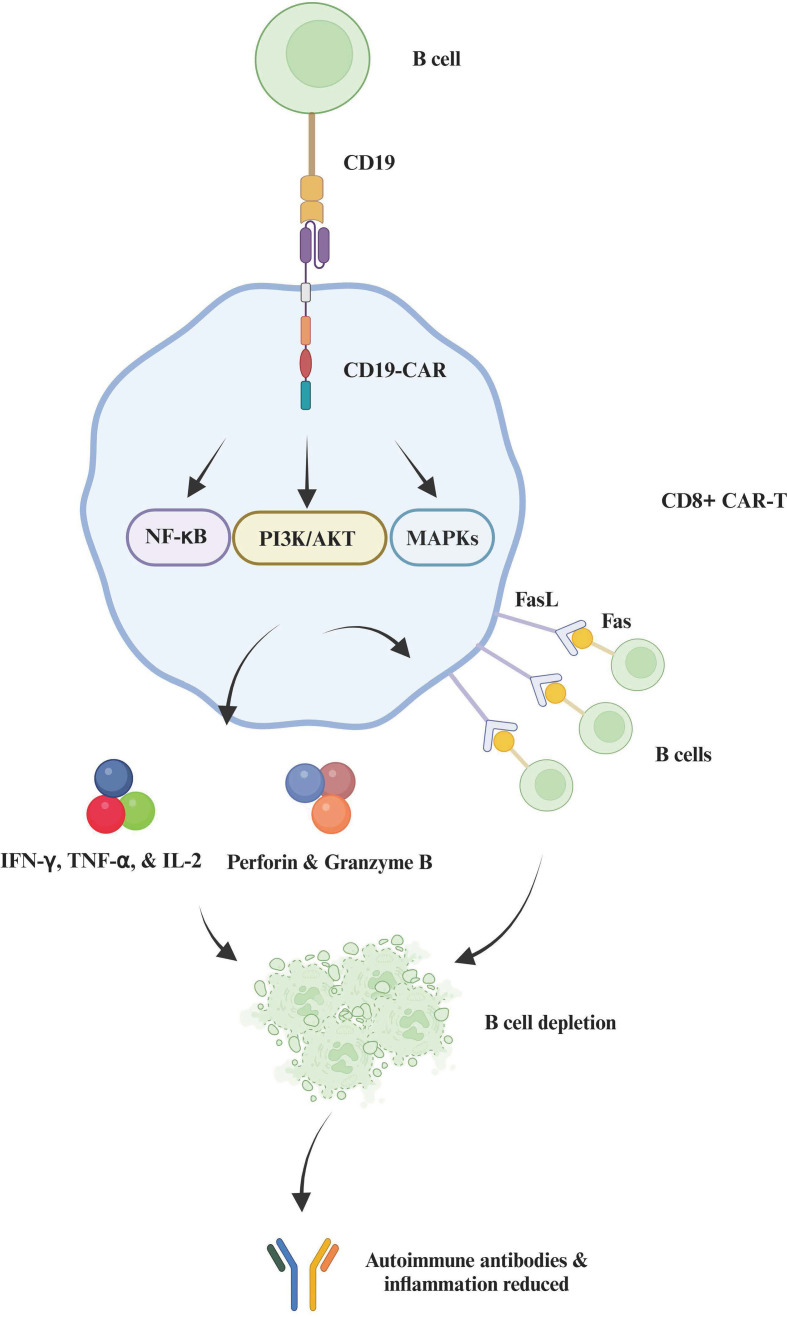
Interaction between activated CD8+ CAR-T cells and B cells in autoimmune modulation. This figure depicts the engagement of an activated CD8+ CAR-T cell with a B cell via a CD19-specific CAR. Upon binding, intracellular signaling pathways such as NF-κB, PI3K/AKT, and MAPKs are activated in the CAR-T cell, facilitating cytotoxic responses. The mechanisms involve perforin and granzyme B release, Fas ligand (FasL) expression for apoptosis induction, and cytokine secretion (IFN-γ, TNF-α, IL-2), leading to targeted B cell depletion. This process effectively reduces autoimmune antibodies and inflammation, illustrating a therapeutic approach in autoimmune disease management.

CAR-T cell therapy is showing promise in treating autoimmune diseases like multiple sclerosis (MS), rheumatoid arthritis (RA), and systemic lupus erythematosus (SLE), offering a more targeted approach than traditional immunosuppressants. By depleting B cells, CAR-T therapy has demonstrated potential in halting disease progression in MS and RA, where CD19-positive B cells and synovial fibroblasts are targeted, respectively (**Figure 3**) (49–52).

Allogeneic CAR-T cells have been tested in severe autoimmune conditions like immune-mediated severe myositis and systemic sclerosis, achieving complete B cell depletion and remission with minimal side effects (30).

In SLE, CAR-T cells have eliminated B cells and reduced autoantibodies, leading to clinical remission (53). Significant trials, including BCMA-targeting (54) and YTS109 (55) for refractory SLE with lupus nephritis, showed promising results, with YTS109 achieving remission in all patients by 3 months and sustained disease reduction by 6 months.

Despite challenges in ensuring specificity and avoiding damage to healthy tissues, early-phase trials suggest that CAR-T therapy offers a more effective and targeted approach to autoimmune diseases, representing a major advancement in the field. While early-phase trials in autoimmune diseases demonstrate remarkable remission rates, the long-term consequences of sustained B-cell depletion remain uncertain, including hypogammaglobulinemia and infection susceptibility. Moreover, whether CAR-T induces true immune reset or merely temporary depletion remains unresolved.

#### 7.1.1 B-cell reconstitution kinetics and immune reset

Although CD19-targeted CAR-T therapy induces profound B-cell aplasia, increasing attention has focused on the kinetics and qualitative nature of B-cell reconstitution following depletion. Emerging clinical observations in autoimmune diseases such as SLE suggest that B-cell recovery typically occurs between 3 to 12 months post-infusion, but the re-emerging B-cell compartment may differ immunologically from the pre-treatment repertoire (56, 57).

Notably, early reconstituted B cells are often enriched for naïve and transitional subsets, with reduced frequencies of class-switched memory B cells and autoreactive clones. This phenomenon has led to the concept of an “immune reset,” whereby autoreactive B-cell networks are disrupted and replaced by a more tolerant repertoire (58). However, the durability of this reset remains uncertain, and long-term longitudinal immune profiling is required to determine whether pathogenic clones re-emerge over time.

#### 7.1.2 Long-lived plasma cells and antigen escape limitations

A major biological limitation of CD19-targeted CAR-T therapy in autoimmune diseases is the persistence of long-lived plasma cells (LLPCs), which typically lack CD19 expression. These cells reside in protective niches within bone marrow and inflamed tissues and continuously secrete pathogenic autoantibodies independent of circulating B-cell populations (59).

Because LLPCs do not express CD19, they are not eliminated by conventional CD19 CAR-T therapy. This may partially explain residual autoantibody persistence in certain patients despite complete peripheral B-cell depletion. Targeting alternative markers such as BCMA (B-cell maturation antigen), which is expressed on plasma cells, has therefore emerged as a complementary strategy (60). Dual CD19/BCMA-targeting CAR constructs or sequential targeting approaches may enhance depletion of autoreactive plasma cell reservoirs and improve durability of remission (61).

#### 7.1.3 Regulatory CAR-T cells and immune tolerance engineering

While most CAR-T strategies rely on cytotoxic CD8^+^ effector T cells to eliminate pathogenic targets, an alternative paradigm involves engineering regulatory T cells (Tregs) with CAR constructs to induce antigen-specific immune tolerance rather than cellular depletion.

CAR-Tregs are designed to recognize tissue- or disease-specific antigens and suppress immune responses locally through mechanisms including IL-10 and TGF-β secretion, CTLA-4–mediated costimulatory blockade, and metabolic modulation. In autoimmune settings, CAR-Tregs may provide a more physiological approach by restoring immune balance instead of inducing broad lymphocyte depletion (62).

Preclinical models have demonstrated that CD19-targeted CAR-Tregs can suppress B-cell–mediated pathology without inducing cytokine release syndrome or systemic immunosuppression (63). Additionally, HLA-A2–specific CAR-Tregs are being explored in transplantation settings to promote graft tolerance while avoiding generalized immune suppression (64).

Compared with cytotoxic CAR-T cells, CAR-Tregs present unique challenges, including phenotypic stability, prevention of lineage conversion into effector T cells, and ensuring sustained suppressive function in inflammatory environments. Advances in FOXP3 stabilization, epigenetic reinforcement, and safety-switch incorporation may enhance clinical feasibility (65).

The development of CAR-Treg platforms represents a conceptual shift from immune ablation toward precision immune reprogramming, potentially offering safer long-term strategies for autoimmune and inflammatory diseases.

#### 7.1.4 Risk considerations: hypogammaglobulinemia, infection, durability, and relapse

Although CAR-T therapy has demonstrated remarkable efficacy in refractory autoimmune diseases, systematic risk assessment remains essential.

CD19- and BCMA-targeted CAR-T therapy frequently induces prolonged B-cell aplasia, resulting in reduced IgG levels. In some patients, hypogammaglobulinemia persists for months and may require immunoglobulin replacement therapy (66). Deeper plasma cell targeting strategies may increase this risk, necessitating regular monitoring of immunoglobulin levels.

Infection susceptibility is temporally dynamic. Early infections are often related to lymphodepletion and transient cytopenias, whereas late infections correlate with sustained B-cell aplasia and low IgG levels (67). Although current autoimmune cohorts report manageable infection rates, long-term real-world data remain limited. Risk-adapted prophylaxis and immunoglobulin support may be required in selected patients.

Emerging data suggest that some patients achieve sustained remission beyond 12–24 months, potentially reflecting an “immune reset” characterized by naïve-dominant B-cell reconstitution (57). However, durability remains heterogeneous. Relapse may result from re-emergence of autoreactive clones, persistence of CD19-negative long-lived plasma cells, limited CAR persistence, or incomplete tissue depletion.

### 7.2 Infectious diseases

CAR-T cell therapy is expanding beyond its traditional use in hematologic cancers to address chronic viral infections like HIV, hepatitis B, and SARS-CoV-2, which evade immune responses due to their ability to mutate (68–70). In HIV, CAR-T cells targeting the virus’s CD4-binding site aim to eliminate infected cells and create HIV-resistant cells. While early trials show feasibility, the long-term efficacy remains under investigation (71–73). A clinical trial involving bNAb-derived CAR-T cells targeting the CCR5 co-receptor in HIV-1 patients demonstrated a significant reduction in viral RNA and proviruses, suggesting the potential for a functional cure by selectively eliminating virus-harboring cells (74).

In hepatitis B and SARS-CoV-2, CAR-T cells targeting viral antigens aim to clear infections and provide lasting immunity, potentially overcoming the limitations of traditional vaccines and antiviral treatments (75–80). However, the rapid mutation rates of these viruses pose challenges in maintaining the effectiveness of CAR-T therapies. Additionally, off-target effects remain a concern, necessitating further optimization of CAR-T cell engineering to improve specificity and minimize risks.

Viral mutation and reservoir heterogeneity represent major biological barriers to durable CAR-T efficacy in chronic infectious diseases. Unlike cancer, viral persistence often involves latent reservoirs that may not uniformly express target antigens, thereby limiting CAR-T recognition and long-term durability. In addition, rapid antigenic evolution may facilitate immune escape, particularly in single-target CAR strategies.

Despite compelling preclinical rationale, the clinical development of CAR-T therapy for chronic viral infections remains in its infancy. To date, no study has demonstrated durable elimination of viral reservoirs or consistent functional cure in humans. Unlike cancer, viral persistence is driven by latency, anatomical sanctuary sites, and dynamic antigen modulation. Latently infected cells often express minimal or no viral antigen, rendering them invisible to CAR-mediated targeting. Furthermore, selective pressure from single-antigen CAR strategies may accelerate viral escape through mutation (74).

In addition to biological barriers, safety considerations are amplified in infectious disease contexts, where patients may otherwise be clinically stable under suppressive antiviral therapy. The risk tolerance for cytokine release, neurotoxicity, or off-target immune damage is therefore substantially lower than in refractory malignancy.

Consequently, while CAR-T platforms offer an innovative conceptual framework for reservoir targeting, their translation into curative antiviral strategies remains speculative and will require multi-specific targeting, latency-reversal integration, and rigorous long-term clinical validation.

## 8 Control mechanisms in CAR-T cell therapy

**Figure 4:**
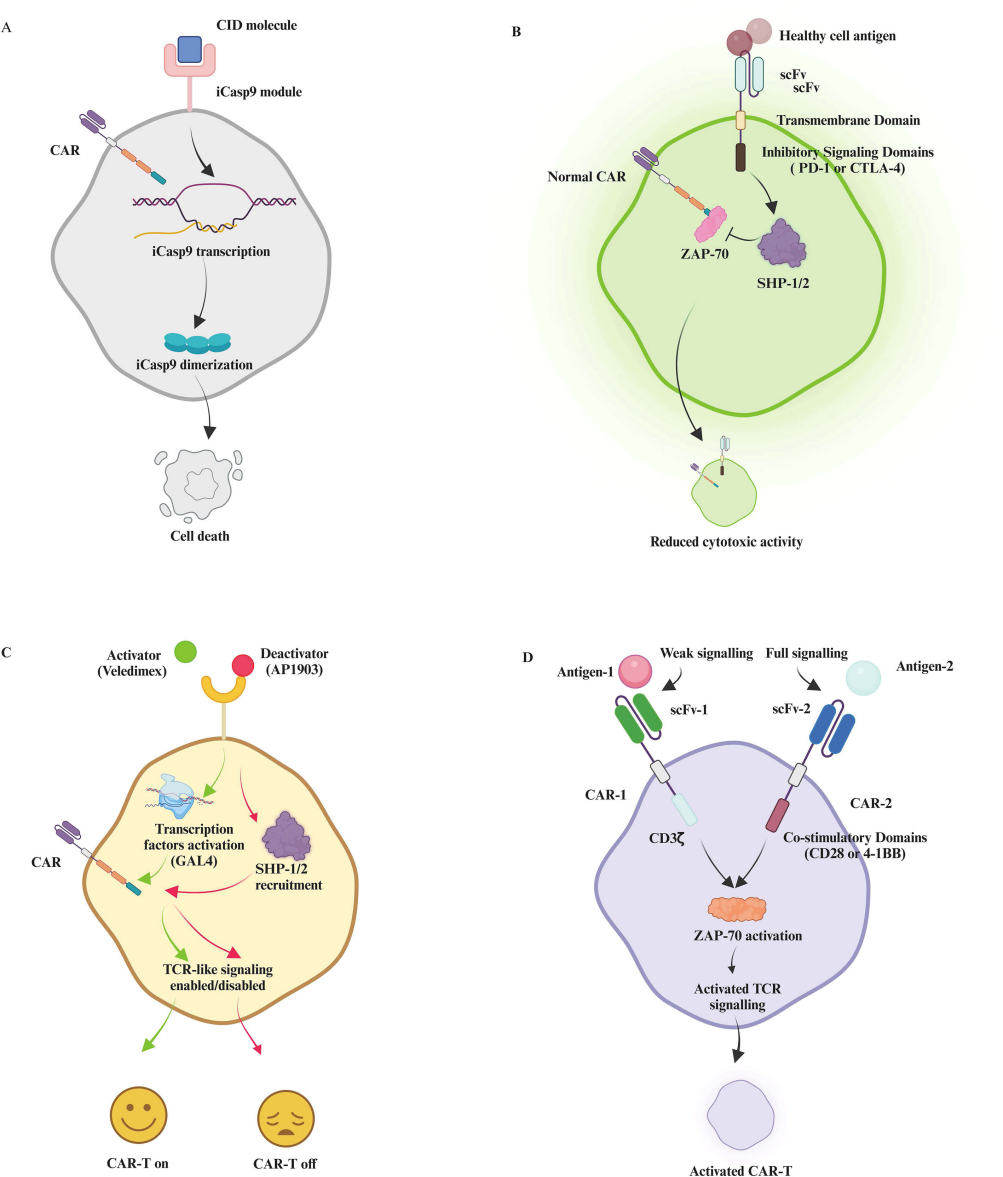
Advanced control mechanisms in CAR-T cell therapy. This detailed illustration provides an overview of advanced regulatory strategies in CAR-T cell therapy aimed at enhancing safety, specificity, and control. **(A)** Showcases the iCasp9 suicide gene system, where the introduction of a chemically inducible dimerizer (CID) molecule leads to the transcription and dimerization of the iCasp9 module within CAR-T cells, ultimately triggering apoptosis to eliminate these cells during adverse reactions. **(B)** Depicts the inhibitory mechanism of iCARs that target antigens on healthy cells, utilizing intracellular domains from PD-1 or CTLA-4 to recruit SHP-1 and SHP-2 phosphatases, which inhibit ZAP-70, thereby reducing cytotoxic activity and preventing damage to non-cancerous cells. **(C)** Illustrates an ON/OFF switch mechanism in CAR-T cell operations, where binding of the activator ligand (Veledimex) to its receptor triggers GAL4-mediated transcription factor activation, promoting TCR-like signaling. This includes ZAP-70 phosphorylation, PI3K/AKT activation, MAPK/ERK pathways, and transcription factor activation of NFAT, NF-κB, and AP-1, collectively enabling cellular activation. Conversely, the deactivator ligand (AP1903) recruits SHP-1/2 to suppress these signaling pathways, effectively turning the CAR-T cell ‘off’. **(D)** Explains the AND-Gate strategy employing dual receptors: CAR-1 with CD3ζ activates initial signaling upon binding to Antigen-1, including partial ZAP-70 activation, while CAR-2, equipped with co-stimulatory domains (CD28 or 4-1BB), requires binding to Antigen-2 for full ZAP-70 activation. This dual recognition ensures potentiated downstream TCR signaling, enhancing therapy efficacy while minimizing off-target effects. iCasp9, Inducible Caspase-9; CID, Chemically Inducible Dimerizer; PD-1, Programmed Death-1; CTLA-4, Cytotoxic T-Lymphocyte Antigen-4; SHP-1/SHP-2, Src Homology 2 Domain-containing Phosphatase-1/2; PI3K, Phosphoinositide 3-Kinase; AKT, Protein Kinase B; ERK, Extracellular Signal-Regulated Kinase; GAL4, Yeast Transcriptional Activator Protein Gal4; AP-1, Activator Protein 1.

As CAR-T cell therapy extends beyond oncology and into other medical domains, ensuring the safety, specificity, and effective regulation of these engineered T cells is crucial. Various control mechanisms have been developed to address the biological and technical challenges associated with CAR-T therapies. Control mechanisms like the iCasp9 suicide gene (81–83), inhibitory CARs (iCARs) (84–87), ON/OFF switch systems (88–90), and AND-Gate strategies (91–93) help mitigate these risks by enhancing specificity and regulating activity (**Figure 4**). Although safety switches enhance control, they may introduce signaling interference, increased construct complexity, and regulatory hurdles. Additionally, multi-component CAR designs may reduce transduction efficiency and increase manufacturing variability. Therefore, the field must balance safety modularity with construct simplicity.

## 9 Visionary perspectives

### 9.1 Future directions

#### 9.1.1 Scalable allogeneic platforms and manufacturing standardization

While gene-edited universal CAR-T strategies have demonstrated feasibility, their long-term impact will depend less on proof-of-concept genome disruption and more on scalable manufacturing, durability, and immune persistence. Future progress will likely focus on optimizing standardized cell sources such as induced pluripotent stem cells (iPSCs), improving immune evasion without excessive genomic complexity, and balancing persistence with safety (94–97).

Emerging approaches such as glycan-mediated immune shielding, controlled cytokine armoring (e.g., IL-15), and locus-specific CAR integration may enhance product consistency while limiting genomic instability (98). Importantly, the next phase of universal CAR-T development will require harmonized quality control standards, reproducible manufacturing workflows, and improved strategies to prevent host immune clearance (99).

Thus, rather than representing a single engineering solution, universal CAR-T platforms may evolve into modular, scalable systems that integrate immune evasion, persistence control, and safety regulation within clinically practical framework.

#### 9.1.2 Precision genome engineering and functional optimization

The refinement of genome-editing technologies is central to next-generation CAR design. CRISPR systems are being utilized to precisely integrate CAR genes into specific loci, reducing the risk of oncogenic transformation associated with random insertion methods (100, 101).

Another innovative platforms like CELLFIE are enhancing CAR-T cell therapy through CRISPR-based gene editing. CELLFIE enables genome-wide screens in primary CAR-T cells, identifying key genes like RHOG and FAS that enhance CAR-T cell efficacy. *In vivo* validation in leukemia models has shown that these gene knockouts improve CAR-T cell function across various CAR designs and patient samples (102). These advancements offer a promising approach to optimize CAR-T therapies and improve clinical outcomes.

#### 9.1.3 Synthetic immune engineering

Synthetic biology has introduced conceptual frameworks for engineering immune cells with programmable and modular functions. In preclinical settings, synthetic gene circuits have been used to regulate activation thresholds, implement logic-gated antigen recognition, and integrate environmental sensing into cellular responses (103–105). These advances suggest the possibility of constructing more precisely controllable CAR-based systems.

However, the development of fully synthetic immune architectures remains largely experimental. Major challenges include ensuring genomic stability, preventing unintended signaling interactions, maintaining phenotypic stability *in vivo*, and satisfying regulatory safety requirements for increasingly complex constructs. At present, incremental strategies—such as locus-specific CAR integration, cytokine armoring, and logic-gated receptor design—are more likely to yield near-term clinical translation than fully synthetic immune platforms.

Thus, while synthetic immune engineering represents a promising conceptual direction, its clinical implementation will require substantial technological validation and long-term safety evaluation.

#### 9.1.4 Artificial intelligence–driven design

Computational modeling and machine learning approaches are increasingly being applied to CAR-T cell development. AI-based methods can assist in antigen epitope prediction, CAR binding affinity optimization, and analysis of high-dimensional single-cell datasets to better characterize CAR-T phenotypes (106–109). These tools may enhance construct screening efficiency and inform rational design strategies.

Nevertheless, AI models remain dependent on high-quality training datasets and experimental validation. Predictions derived from computational systems require rigorous biological confirmation, and current algorithms cannot fully capture the complexity of *in vivo* immune dynamics. Rather than replacing empirical development pipelines, AI currently functions as a complementary optimization tool that may streamline hypothesis generation and reduce iterative design cycles.

Accordingly, while AI-assisted design holds promise for improving CAR construct refinement, its role should be viewed as supportive rather than transformative at the present stage of clinical development.

#### 9.1.5 Expanding the CAR platform beyond T cells

Future CAR strategies are also expected to extend beyond T-cell–centric approaches. CAR-NK cells represent a complementary effector platform with strong potential for standardized, cryopreserved manufacturing, particularly through iPSC-derived master cell banks. Their scalability supports rapid deployment models suited for broader clinical accessibility (110).

Advances in synthetic biology, cytokine armoring (e.g., IL-15), gene editing, and metabolic programming are likely to further enhance NK cell persistence and function within suppressive tumor microenvironments (111). Moreover, sequential or combinatorial administration of CAR-T and CAR-NK therapies could leverage complementary immune dynamics to reduce relapse and mitigate antigen escape (112). For example, CAR-NK cells may provide rapid cytoreduction with limited toxicity, followed by CAR-T cells to establish durable adaptive immunity.

Ultimately, CAR-based therapy may evolve into a modular immune engineering ecosystem in which the choice of effector cell type is guided by disease biology, therapeutic urgency, and safety considerations rather than reliance on a single dominant modality.

### 9.2 Global implications

The broader implementation of CAR-T therapy presents significant logistical and economic challenges. Current manufacturing processes require specialized infrastructure, highly trained personnel, and complex supply chains, limiting accessibility outside major tertiary medical centers (113, 114). Cost remains a major barrier, particularly in low- and middle-income regions.

Efforts to improve scalability—including allogeneic platforms, automated manufacturing systems, and *in vivo* engineering approaches—may reduce structural barriers over time. However, achieving equitable global access will require coordinated regulatory frameworks, sustainable reimbursement models, and international collaboration to expand technical capacity.

While CAR-based therapies may eventually become more widely accessible, substantial infrastructural, economic, and policy challenges must be addressed before broad global deployment can be realized.

## 10 Limitations and process development bottlenecks in CAR-T cell therapy

### 10.1 Autologous manufacturing variability and logistical constraints

Autologous CAR-T therapy is inherently individualized, relying on patient-derived T cells that often vary in quality due to prior treatments, disease burden, or immune exhaustion. This variability affects transduction efficiency, expansion capacity, and final product potency, contributing to inconsistent manufacturing outcomes. The multi-step production workflow—encompassing leukapheresis, activation, gene transfer, expansion, quality control testing, cryopreservation, and reinfusion—introduces multiple potential points of delay or failure. Prolonged vein-to-vein times may compromise patients with rapidly progressing disease. Moreover, the personalized nature of autologous production limits scalability and contributes substantially to high treatment costs.

### 10.2 Allogeneic platforms: immunological and gene-editing challenges

Allogeneic and universal CAR-T strategies seek to improve scalability through off-the-shelf availability but introduce additional biological complexities. Prevention of GVHD disease requires disruption of endogenous T-cell receptor signaling, while host immune rejection necessitates HLA modification or immune-evasive engineering. These multiplex gene-editing strategies increase manufacturing complexity and raise concerns regarding genomic stability, off-target effects, and regulatory scrutiny. In addition, reduced *in vivo* persistence due to host immune clearance remains a major limitation for many allogeneic products.

### 10.3 Vector supply and gene delivery limitations

Viral vector production remains a critical bottleneck across both platforms. Lentiviral and retroviral systems require specialized Good Manufacturing Practice facilities, are costly to produce, and have limited global manufacturing capacity. These constraints restrict clinical scalability and commercial deployment. Although non-viral approaches such as CRISPR-mediated targeted integration, transposon systems, and mRNA delivery are under development, they must balance efficiency, durability, and safety while meeting regulatory standards.

### 10.4 Quality control and standardization barriers

CAR-T products are biologically heterogeneous living therapies, making standardization challenging. Variability in T-cell subsets, CAR expression levels, and functional potency complicates release criteria and cross-trial comparisons. Furthermore, *in vitro* potency assays do not consistently predict *in vivo* persistence or clinical response. The lack of harmonized global standards for quality control and long-term monitoring adds further complexity to regulatory approval and commercialization.

### 10.5 Construct complexity in solid tumor applications

The development of CAR-T therapies for solid tumors often requires additional genetic modifications, such as cytokine armoring, checkpoint disruption, or logic-gated designs to overcome the immunosuppressive tumor microenvironment. Increasing construct complexity can reduce transduction efficiency, complicate manufacturing reproducibility, and intensify regulatory oversight. These factors further slow clinical translation in solid tumor settings.

### 10.6 Economic and infrastructural constraints

CAR-T therapy requires specialized facilities, trained personnel, and coordinated supply chains, limiting accessibility in resource-constrained settings. High upfront costs and evolving reimbursement models pose additional barriers to widespread adoption. Addressing these systemic challenges will be essential to ensure equitable global access.

### 10.7 Core unresolved biological bottlenecks in CAR-T therapy

Despite major clinical advances, fundamental biological bottlenecks continue to limit the durability and broader applicability of CAR-T therapy. Therapeutic efficacy remains vulnerable to antigen heterogeneity and escape, as target cells may downregulate or mutate antigen expression under selective pressure. Sustained CAR signaling can induce T-cell exhaustion and functional instability, compromising long-term persistence. In addition, immunosuppressive microenvironments and anatomical sanctuary sites restrict effective expansion and target engagement. A critical persistence–safety trade-off persists, as prolonged CAR-T activity may lead to chronic cytopenias or off-tissue toxicity. In allogeneic platforms, balancing GVHD prevention with avoidance of host rejection further complicates durable engraftment. Together, these biological constraints—beyond manufacturing challenges—define the central barriers to achieving safe and durable CAR-T therapies.

## 11 Conclusion

CAR-T cell therapy has revolutionized cancer treatment and shows promise for a wide range of conditions beyond oncology, including autoimmune diseases, viral infections, and solid tumors. Despite significant progress, challenges like cytokine release syndrome and graft-versus-host disease remain. Emerging strategies, such as universal and *in vivo* CAR-T cells, aim to enhance safety and accessibility. The integration of technologies like CRISPR and artificial intelligence will further improve CAR-T’s efficacy and application, with the potential to transform treatment paradigms across various diseases. Sustained technological refinement, regulatory harmonization, and interdisciplinary collaboration will be essential to address unresolved biological and logistical barriers and to enable broader clinical integration.
